# The Importance of the Double Product in the Six-Minute Walk Test to Predict Myocardial Function

**DOI:** 10.1155/2018/3082690

**Published:** 2018-07-04

**Authors:** Elżbieta Domka-Jopek, Andrzej Jopek, Agnieszka Bejer, Ewa Lenart-Domka, Grzegorz Walawski

**Affiliations:** ^1^Institute of Physiotherapy, Faculty of Medicine, The University of Rzeszow, Rzeszów, Poland; ^2^Specialist District Hospital, Leżajsk, Poland; ^3^The Holy Family Specialist Hospital, Rudna Mała, Poland; ^4^The Child and Adolescent Neurological Rehabilitation Department, Clinical Hospital No. 2, Rzeszów, Poland

## Abstract

**Introduction:**

The Six-Minute Walk Test (6MWT) is a widely used test to measure the physical performance of patients to assess the effectiveness of treatment, to qualify for rehabilitation, and to evaluate its effects..

**Aim:**

This paper focuses on the assessment of the growth of a double product (DP) during the 6MWT and its diagnostic value in the assessment of patients with heart failure.

**Material and Methods:**

The paper has retrospective character. We analyzed medical records of 412 patients hospitalized for cardiac reasons, in whom a 6MWT was performed. The patients were divided into two groups: one with diagnosed heart failure and a control group.

**Results:**

The patients with diagnosed heart failure, compared to the control group, were characterized by a shorter walking distance and greater DP increase at equal walking intervals. After distinguishing the group with the preserved and decreased left ventricle ejection fraction, the value of the DP increase was still higher compared to the control group. The mean DP increase corresponding to one meter of walk was the only one that correlated negatively with the left ventricular ejection fraction.

**Conclusion:**

The assessment of the increase of the DP during the march test seems to be a better parameter reflecting the efficiency of the myocardium from the distance of the march.

## 1. Introduction

The Six-Minute Walk Test (6MWT) is a widely used test to measure physical capacity of patients [1, 2], to assess the effectiveness of treatment, to qualify for rehabilitation, and to assess its effects. The advantages of this test include its simplicity, low costs, and lack of equipment requirements. There is a work in which the authors argue that it is possible to administer the 6MWT independently, only with the help of a suitable smartphone application [3]. At the same time, this test is safe and well tolerated by patients [4–6]. Although the circulatory-respiratory exercise test has remained to be the classic cardiac stress test used in patients with cardiovascular disease, the 6-Minute Walk Test is also very often used to assess physical performance, as a correlation was found between the 6MWT distance and the maximum sustainable load [7] or peak oxygen uptake (maxVO2) in the exercise test [8]. It should be emphasized that, unlike the classic exercise test, the 6MWT does not detect any changes in the ECG during exercise (e.g., arrhythmia, ischemia), and it does not recognize the cause of breathlessness during exercise, nor does it assess the causes or mechanisms of limiting physical effort [9]. When performing a classic exercise test is impossible (lack of appropriate equipment, qualified personnel), and the 6MWT can be very helpful to assess the physical performance of patients. Finally, taking into account the aforementioned advantages (low costs, simplicity of the study), the 6MWT may become a population-based study that allows assessing the physical capacity of large groups of patients.

Apart from the obvious role of the 6MWT in the assessment of physical capacity of patients with heart disease, this test was also used to assess the improvement of patients taking part in modern treatment programs [10, 11], undergoing cardiac surgery [12, 13], or participating in cardiac rehabilitation programs [6, 7, 14]. The 6MWT can be important in choosing the right treatment procedures. It can be used, for example, in the qualification of patients with heart failure for ICD implantation or for amiodarone therapy [15].

Numerous papers indicate the importance of the walk test in predicting the risk of future hospitalizations and the mortality risk in patients with cardiovascular disease. The beneficial prognostic role of the 6MWT was demonstrated in patients with heart failure and low left ventricular ejection fraction [16–18]. Similar relationships have been demonstrated for patients with heart failure and preserved left ventricular diastolic function [19]. A meta-analysis of patients with dilated cardiomyopathy showed a positive correlation between the walking distance and the number of deaths due to cardiac reasons. The reverse relationship was found between the distance in the 6MWT and the number of transplants in this group of patients. In patients with stable coronary heart disease, a relationship was observed between the distance in the corridor walk test and the risk of cardiovascular events [20]. Finally, in patients with asymptomatic and symptomatic aortic stenosis, the shorter distance in the walk test was burdened with an increased risk of death due to cardiac reasons [21, 22]. There are also papers that do not support the above role of 6MWT in predicting the risk of cardiovascular events and deaths in patients with heart failure [8], including patients undergoing CABG [23]. It is not completely clear if the 6MWT is equivalent to the exercise test in terms of prognostic significance of both tests. Some of the papers indicate similar prognostic power [16, 20], while others do not show such equivalence [8].

Wide use of the 6MWT in clinical practice in patients with cardiovascular diseases, as well as some discrepancies in the assessment of its usefulness in prognosis in this group of patients, requires further research to determine the significance of this diagnostic method. It is interesting to analyze, apart from the distance of the march, the parameters obtained from the 6MWT in patients with symptoms of heart failure.

We hope that the change in the double product analyzed by us during the march test will contribute to the increase in the significance of the 6MWT for the clinical evaluation of patients.

## 2. Aim

This paper focuses on the assessment of the growth of a double product during the walk test and its diagnostic value in the assessment of patients with heart failure. The double product corresponding to the product of heart rate and systolic blood pressure has a well-established position as a parameter expressing the load on the heart muscle. As an easily accessible parameter, it has been used in clinical trials for many years [24] and still proves its diagnostic [25] and prognostic [26, 27] usefulness.

## 3. Material and Methods

The paper has retrospective character. Medical records of 412 patients hospitalized in the Department of Cardiology of the Hospital in Leżajsk (a town in the southeast of Poland) in 2010–2017 were analyzed.

Inclusion criteria are as follows:

- sinus rhythm during the walk test and echocardiography
- echocardiography and 6MWT performed during one hospitalization.

Exclusion criteria are as follows:

- atrial fibrillation
- stimulatory rhythm.

Echocardiography was performed with Vivid 5 apparatus. The ejection fraction was calculated using the Simpson method [28].

Patients were divided into two groups: a group of patients with diagnosed heart failure and a control group. The control group consisted of patients hospitalized for other reasons (i.e., due to hypertension, diagnosis of syncope, and paroxysmal arrhythmias). In the second stage, patients with preserved left ventricular ejection fraction (EF≥50%, HFpEF) were isolated from patients with heart failure and reduced left ventricular systolic function (EF <50%). The adopted values of the fractions are reported in the literature as differentiating the patients with heart failure [29, 30].

The 6MWT was performed according to the standard walk test protocol [9]. The results included walking distance (in meters), resting and final double product (calculated as the product of the heart rate [number of beats/min], and systolic blood pressure [mmHg], respectively, before and immediately after the test), double product gain (understood as the difference between the final double product and resting double product), and symptoms reported by the patient during exercise. In addition, we considered it appropriate to calculate the value of the average increase in double product per one meter of march (the quotient of the double product increase to the distance during the test). The lack of the imposed gait rate, resulting from the assumptions of the walk test, makes the patients overcome any distance (not necessarily limited by symptoms). Therefore, it is difficult to compare such patients in terms of double product growth. In addition, a number of parameters such as muscle strength, balance disorders, mood, and overall health have a proven effect on the walking distance achieved by the patients [2]. It seems that the comparison of patients in terms of the growth of a double product made during the analogous walking distance should systematize the obtained results.

The exclusion of patients with atrial fibrillation and stimulatory rhythm from the study resulted from another way of promoting myocardial excitation, which in our opinion has an impact on the heart rate, and therefore, the value of the double product.

### 3.1. Data Analysis

Data are presented in the form of arithmetic means ± standard error of the mean (M ± SEM). Correlation tests of Person and Spearman were used in the correlation analysis. In case of the normal distribution of variables, Student's* t*-test was used for unrelated or related variables distribution. To compare the means in more than two groups, the analysis of variance nonparametric test was used (Kruskall-Wallis test). Equality of variance was evaluated by the Fischer test. In each case p values <0.05 were considered statistically significant.

## 4. Results

Populations of the patients with heart failure and the control group were of similar age. They were significantly different in terms of body weight, diabetes, and creatinine (patients with heart failure were more obese; they had higher incidence of diabetes and showed increased blood creatinine). They did not differ in the level of hemoglobin, TSH, and the frequency of diagnosed hypertension and dyslipidemia (Tables 1(a) and 1(b)). These groups were significantly different in terms of the incidence of symptoms of heart failure during the 6MWT (Table 1(d)). Both patients populations did not differ significantly in the use of drugs that could affect heart rate and blood pressure (data not shown).

**Figure 1:**
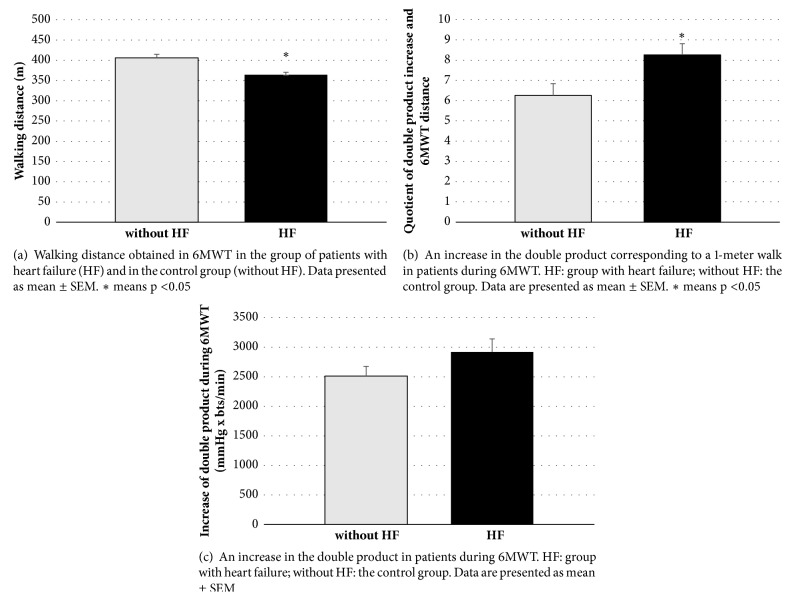


Patients with diagnosed heart failure covered a shorter distance in the 6MWT compared to the control group (Figure 1(a)). In this group, the increased heart load during exercise was also assessed by the increase in the double product per meter (double product quotient and walking distance, Figure 1(b)). Measurement of the double product gain during the entire exercise (i.e., evaluated at different walking distances) showed a trend of a larger increase in the group with heart failure, but this change was not statistically significant (Figure 1(c)).

**Figure 2:**
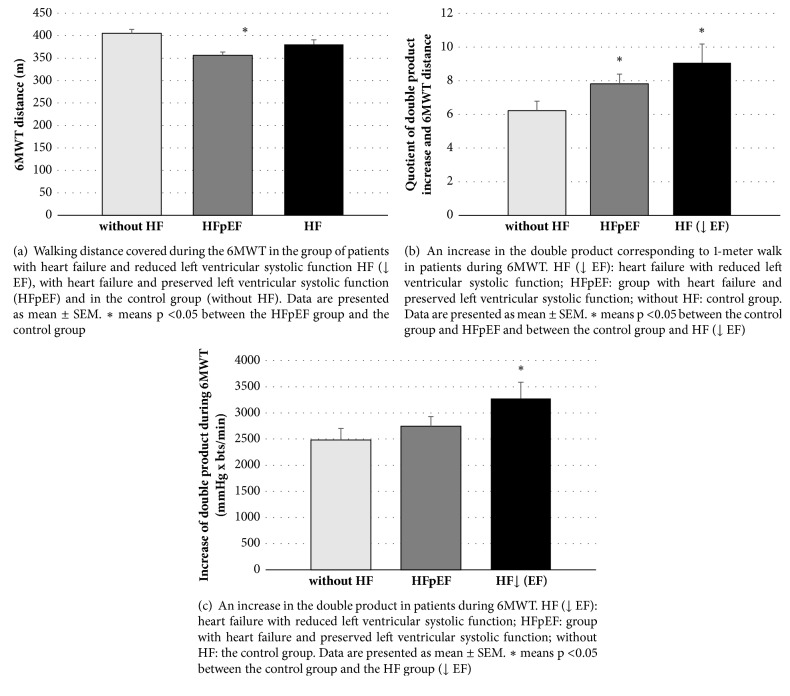


**Figure 3:**
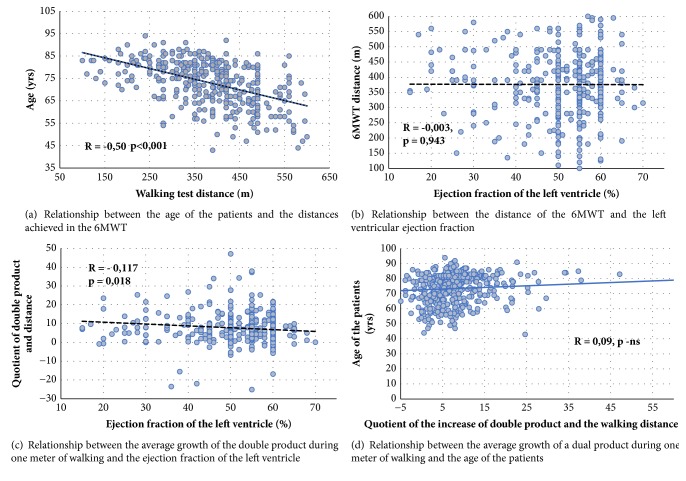


After dividing the patients with heart failure into two groups: with preserved and decreased left ventricular systolic function, it turned out that the significant shortening of the walking distance was characteristic only for patients with HFpEF (Figure 2(a)). The patients in this group were older and characterized by a higher BMI than other groups (Table 1(c)). Significant influence of age on the distance achieved seems to explain the differences in the described groups (Figure 3(a)).

In the analysis of changes in the double product during the walk test, a larger increase in the double product was found in the group with reduced left ventricular ejection fraction compared to the control group (Figure 2(c)). The mean double product growth corresponding to 1 meter of walk (quotient of double product and walking distance) was the highest in the group with HF (↓ EF), it had an intermediate value in the group with HFpEF, and these differences in both groups were significant in relation to the control group (Figure 2(b)). Importantly, there was no significant relationship between the age of the patients and the increase in the double product during exercise (Figure 3(d)).

Analyzing the correlation between left ventricular ejection fraction and parameters obtained from the walk test, it was shown that only the mean double product growth corresponding to 1 meter walk (the quotient of the double product and the walking distance) correlates negatively with the left ventricle ejection fraction (Figures 3(b) and 3(c)).

Assessment of the quotient of a double product during walking and walking distance covered seem to be the best parameter reflecting the efficiency of the myocardium.

**Figure 4:**
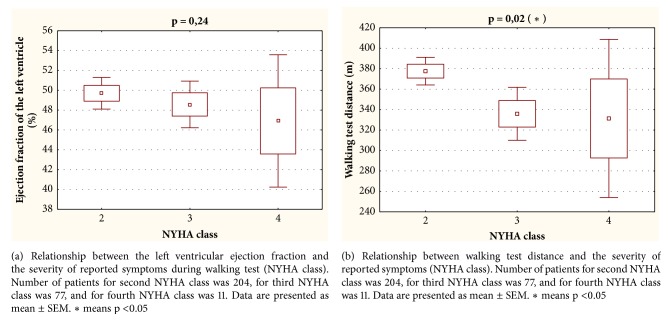


We could not show a significant relationship between the left ventricular ejection fraction and the severity of reported symptoms (NYHA class, Figure 4(a)). However, a significant negative correlation was found between the size of the walking distance and severity of symptoms (NYHA class, Figure 4(b)).

**Figure 5:**
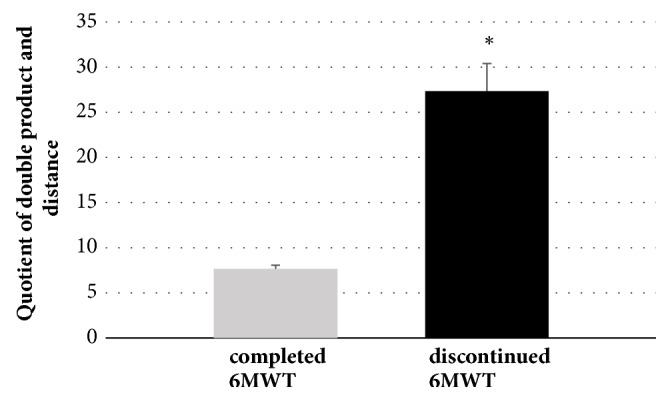
Increase of double product corresponding to 1-meter walk during 6MWT in patients who completed and discontinued the test (<6min). Data presented as a mean ±SEM. *∗* means p<0,05.

In the last stage, patients who completed the 6MWT and patients who had to stop the test due to severe symptoms were compared. In the group of patients who completed the march, there was a significantly lower increase in the double product in relation to a 1-meter walk (Figure 5). These groups did not differ in terms of age, BMI, and TSH (data not shown).

It should be emphasized that none of the patients subjected to the 6MWT showed significant complications requiring the use of above-standard medical intervention.

## 5. Discussion

The study was based on data obtained from 412 patients hospitalized for cardiac reasons in a district hospital. The meaning of the 6MWT in the context of the assessment of patients for the incidence of heart failure was analyzed. This paper focuses on the evaluation of a double product calculated in patients during the 6MWT. It is an easily accessible parameter with a proven role in the assessment of myocardial load [31].

The analysis of the available literature shows, however, that few authors evaluate the changes of this parameter during the 6MWT, their conclusions are based only on the evaluation of the distance of the walk. Among the studies also taking into account the double product, it was shown that, in patients with chronic obstructive pulmonary disease, the assessment of the double product correlated better with the results of a standard exercise test than the walking distance [32]. Other authors analyzing the effectiveness of cardiac rehabilitation in patients after CABG showed that, in the group of patients participating in the rehabilitation, the distance covered during the test and the maximum double product increase [33]. Increasing the double product as the exertion intensifies seems natural. The value of a double product is directly dependent on the heart rate, which normally increases with the exertion [34].

There are several studies evaluating the double product during the walk test in patients after a stroke. Eng et al. have not shown correlations between the double product and the walking distance in these patients [35]. In our study, no correlation between the growth of the double product and the distance of the walk has been demonstrated (data not shown). Other authors, in order to assess the physical capacity of the patients after stroke, compared the 6MWT and the 6-minute step test (6MST) [36]. In both tests, the only parameter analyzed was the double product. For obvious reasons, both studies were based on data from very small groups of patients (25 and 12 patients, respectively).

In the presented analysis, the patients with diagnosed heart failure achieved a shorter distance in the 6MWT compared to the control group. The meta-analysis of data obtained from patients with heart failure showed a shorter walking distance in patients with more advanced heart failure, assessed on the basis of the NYHA classification. One limitation, however, was the large variety of walking distances for each class [37]. In our study, after analyzing patients with heart failure for the NYHA class, it turned out that the vast majority (69%) had slight symptoms qualifying them for the NYHA class II. Of course, for understandable reasons, patients in the acute condition (as a rule NYHA class IV) did not have walk tests performed until the clinical condition improved (contraindications for the march test). Therefore, there is a small representation of patients in the NYHA IV class. We could not show a significant relationship between the left ventricular ejection fraction and the severity of reported symptoms (NYHA class, Figure 4(a)). However, a significant negative correlation was found between the size of the walking distance and severity of symptoms (NYHA class, Figure 4(b)). In both situations, the particular NYHA class was connected with large variety of left ventricular ejection fraction or walking distance. Therefore, the results of our research correspond to the above-mentioned work [37]. It is worth noting that the NYHA classification operates with little specific symptoms of heart failure (fatigue, dyspnea, and palpitations during exercise). Such symptoms may result from other diseases and they will decompose differently due to the age of the patients (e.g., the occurrence of tiredness). It is also known that the walking distance strongly correlates with the age of the patients. So, the NYHA classification, despite the undoubted importance in classifying patients in terms of the severity of heart failure, cannot constitute a certain criterion for the occurrence of heart failure.

In the paper devoted to physical capacity of patients with congenital heart disease, a correlation was also found between the severity of heart failure (expressed by BNP level) and the distance of the 6MWT [38].

In the present study, a secondary analysis of patients with heart failure was made after dividing them into groups: with preserved and decreased systolic function of left ventricular ejection fraction. According to current standards, heart failure is not a homogenous unit, but it consists of two components: heart failure with preserved and decreased left ventricular ejection fraction [39]. There is a research analyzing the 6MWT with similar divisions in patients. In this study, however, they focused on the prognostic importance of the walking test distance [8].

In our study, after the division of patients due to the size of the ejection fraction, it turned out that the significant shortening of the walking distance in relation to the control group concerned only patients with preserved left ventricular ejection fraction. Patients in this group were, however, older, which, taking into account the strong influence of age on the distance covered, may explain the differences in the groups described. There are a number of papers in which the negative effect of age on walking distance [6, 40] is described.

Despite a large number of studies using the 6MWT to assess patients with heart failure, we were unable to find a study analyzing the use of double product gain during the test to assess the severity of this condition. However, there are papers that use the concept of a double product in the group of patients with heart failure in the context of the assessment of the effectiveness of cardiac rehabilitation [41, 42]. In case of these studies, the benefits of rehabilitation meant a smaller increase in the double product during exercise in patients participating in rehabilitation. In our work, the presence of heart failure in patients was associated with a greater increase in the double product during the 6MWT compared to the control group. A significant correlation was also found between the size of the increment of the double product with 1-meter walk and the size of the left ventricular ejection fraction. This is a small correlation, which seems understandable given that also patients with heart failure and preserved left ventricular systolic function have a significant increase in the double product compared to the control group. Similar correlation was not found by analyzing the walking distance and left ventricular ejection fraction. Therefore, the assessment of walking distance in the patients with cardiac problems does not appear to provide additional information in terms of cardiovascular system capacity. Also, in other studies, there was no correlation between the measured walking distance and the left ventricular ejection fraction [43, 44]. Interestingly, it seems that the pulse amplitude during the walking test (which partially reflects the double product gain) is a much stronger prognostic factor in patients with heart failure than the distance itself [45].

There is a study in which the authors analyzed the effect of assisted breathing (CPAP) on physical capacity in patients with heart failure [46]. They used the 6MWT to assess physical performance, and one of the parameters studied was the double product at the end of the exercise. The authors of this paper showed that, after the use of assisted breathing, the distance of the walking test increases, which is accompanied by the reduction of the double product. In this study, better oxygenation of the blood had an impact on the reduction of the double product evaluated after the walk test. In our study, a smaller increase in the double product was associated with the lack of heart failure. As other studies show, the occurrence of heart failure is associated with a lower oxygenation of arterial blood during exercise. Therefore, it can be expected that, in patients with heart failure, the increase in the double product should be higher in comparison to patients without heart failure. Confirmation of such assumptions is the result of our work. The existing differences in the methodology of the aforementioned study and ours (in our study the increase in the double product, in the other study the double product at the end of the effort) and the small number of people in the CPAP study (n = 12) limit the possibilities of comparing these results.

Interestingly, in studies demonstrating the clinical usefulness of the double product, the value of double product was often used (RPP reverse) rather than the maximum or resting double product. In our studies, the assessment of the double product increase was best correlated with the echocardiographic results.

The results of our research indicate that the 6MWT is safe in the group of patients with heart failure. Analysis of the double product gain during this study can provide valuable information on exercise tolerance and can provide further information about cardiovascular performance. The assessment of the RPP is a complement to imaging tests, and, in some cases, when it is impossible to perform echocardiography, it may, within certain limits, reflect the degree of cardiovascular function. Of course, we treat our research as a pilot study, requiring confirmation in further observations.

## 6. Conclusion

The assessment of the increase of the DP during the march test seems to be a better parameter reflecting the efficiency of the myocardium from the distance of the march.

## Figures and Tables

**Table 1a:** (a) Groups characteristics in terms of age, body weight, and selected laboratory parameters

	Patients without HF (n=120)	Patients with HF (n=292)	p
Age (yrs)	72 ±0,94	74,1 ±0,58	ns
BMI	29,14 ±0,49	30,38 ±0,31	< 0,05
Hemoglobin (g/dl)	13,54 ± 0,12	13,25 ±0,12	ns
TSH (uUI/l)	1,86 ± 0,26	2,09 ±0,34	ns
Creatinine (g/dl)	0,90 ± 0,02	0,98 ± 0,02	<0,05

**Table 1b:** (b) Groups characteristics in terms of the presence of risk factors for atherosclerosis

	Patients without HF (n=120)	Patients with HF (n=292)	p
Hypertension (% of population)	85	88	ns
Lipid disorders (% of population)	64	58	ns
Diabetes mellitus (% of population)	20	30	< 0,05

**Table 1c:** (c) Groups characteristics in terms of age, body weight, and selected laboratory parameters. Normal font: comparison of groups I and III, **bold**: comparison of groups II and III, *italics*: comparison of groups I and II

	without HF (n=119)	HFpEF (n=192)	HF (↓EF) (n=102)	p
Age (yrs)	72 ±0,94	75,75 ± 0,65	71,49 ± 1,08	*<0,05*; **0,05**
BMI	29,14 ±0,49	31,23 ± 0,38	28,91 ± 0,49	*<0,05*; **<0,05**
Hemoglobin (g/dl)	13,54 ± 0,12	13,22 ± 0,11	13,29 ±0,26	ns
TSH (uUI/l)	1,86 ± 0,26	2,41 ± 0,52	1,55 ± 0,13	ns
Creatinine (g/dl)	0,90 ± 0,02	0,91 ±0,02	1,10 ±0,05	< 0,05; **<0,05**

**Table 1d:** (d) The incidence of symptoms during the walking test

	Patients without HF (n=120)	Patients with HF (n=292)	p
Dyspnea during walking test (% of population)	17,6	28,1	<0,05
Feeling the load during the walking test (average score in Borg scale)	7,74 ± 0,25	8,65 ± 0,19	< 0,05

## Data Availability

The data used to support the findings of this study are available from the corresponding author upon request.
